# Heterozygous APC germline mutations impart predisposition to colorectal cancer

**DOI:** 10.1038/s41598-021-84564-4

**Published:** 2021-03-04

**Authors:** Livia Preisler, Aline Habib, Guy Shapira, Liron Kuznitsov-Yanovsky, Yoav Mayshar, Ilana Carmel-Gross, Mira Malcov, Foad Azem, Noam Shomron, Revital Kariv, Dov Hershkovitz, Dalit Ben-Yosef

**Affiliations:** 1 Wolfe PGD-Stem Cell Laboratory, Racine IVF Unit, Lis Maternity Hospital, Tel-Aviv Sourasky Medical Center, 64239 Tel-Aviv, Israel; 2 Department of Cell and Developmental Biology, Sackler Faculty of Medicine, Sagol School of Neuroscience, Tel-Aviv University, Tel-Aviv, Israel; 3 Department of Gastroenterology, Tel-Aviv Sourasky Medical Center, Tel-Aviv, Israel; 4 Institute of Pathology, Tel-Aviv Sourasky Medical Center, Tel-Aviv, Israel; 5 Present Address: Department of Molecular Cell Biology, Weizmann Institute of Science, Rehovot, Israel

**Keywords:** Cancer models, Gastrointestinal cancer, Tumour-suppressor proteins

## Abstract

Familial adenomatous polyposis (FAP) is an inherited syndrome caused by a heterozygous adenomatous polyposis coli (APC) germline mutation, associated with a profound lifetime risk for colorectal cancer. While it is well accepted that tumorigenic transformation is initiated following acquisition of a second mutation and loss of function of the APC gene, the role of heterozygous APC mutation in this process is yet to be discovered. This work aimed to explore whether a heterozygous APC mutation induces molecular defects underlying tumorigenic transformation and how different APC germline mutations predict disease severity. Three FAP-human embryonic stem cell lines (FAP1/2/3-hESC lines) carrying germline mutations at different locations of the APC gene, and two control hESC lines free of the APC mutation, were differentiated into colon organoids and analyzed by immunohistochemistry and RNA sequencing. In addition, data regarding the genotype and clinical phenotype of the embryo donor parents were collected from medical records. FAP-hESCs carrying a complete loss-of-function of a single APC allele (FAP3) generated complex and molecularly mature colon organoids, which were similar to controls. In contrast, FAP-hESCs carrying APC truncation mutations (FAP1 and FAP2) generated only few cyst-like structures and cell aggregates of various shape, occasionally with luminal parts, which aligned with their failure to upregulate critical differentiation genes early in the process, as shown by RNA sequencing. Abnormal disease phenotype was shown also in non-pathological colon of FAP patients by the randomly distribution of proliferating cells throughout the crypts, compared to their focused localization in the lower part of the crypt in healthy/non-FAP patients. Genotype/phenotype analysis revealed correlations between the colon organoid maturation potential and FAP severity in the carrier parents**.** In conclusion, this study suggest that a single truncated APC allele is sufficient to initiate early molecular tumorigenic activity. In addition, the results hint that patient-specific hESC-derived colon organoids can probably predict disease severity among FAP patients.

## Introduction

Colorectal cancer (CRC) is the third most frequently diagnosed cancer and the second leading cause of cancer death worldwide^1^. Colorectal tumorigenesis begins with the transformation of normal epithelium to aberrant crypt foci, which then progress to adenoma and proceed to carcinoma^2,3^. This process is closely linked to the stepwise accumulation of multiple genetic and epigenetic aberrations that promote deregulated differentiation and uncontrolled proliferation^4–6^. Inactivation of adenomatous polyposis coli (*APC*), a tumor suppressor gene, has been recognized as a key early event in up to 85% of sporadic CRCs^4,7^. While most cases of CRC are considered sporadic, hereditary CRC syndromes account for 2–5% of all cases^8^. As hereditary CRC syndromes are caused by genes that are also somatically altered in the sporadic form, they have been used as a model to understand the molecular pathogenesis underlying CRC in general^9^. Familial adenomatous polyposis (FAP) is an inherited autosomal-dominant syndrome caused by a germline mutation in the *APC* gene^10^. In accordance with Knudson’s ‘two hit’ hypothesis, FAP patients with a germline APC mutation have a very high risk to acquire an additional somatic APC mutation mainly in the colorectum, that will eventually lead to the development of multiple adenomas, starting at adolescence, with nearly 100% progression to CRC by the age of 40, if left untreated^10–12^. The site of the 'first hit' in the APC tumor suppressor gene has an impact on the type of the 'second hit' that will either cause APC loss of heterozygosity or a truncated protein^13–17^. Moreover, the type and location of this germline mutation are associated with colonic polyp burden, surgical outcome, and the presence of extra-colonic FAP manifestations^13,18^.

Although the role of APC in CRC initiation has been extensively studied^19–24^, it is not yet clear whether a heterozygous APC mutation is sufficient to cause the earliest molecular changes leading to colon carcinogenesis in human. It also remains to be determined if various APC germline mutations differentially affect the initiation and the severity of the developing disease. In the current study, three FAP-human embryonic stem cell (hESCs) lines carrying various human APC germline mutations that were derived in our lab from donated embryos^25,26^, were exploited to study whether a mutation in a single allele of APC is sufficient to alter the molecular and cellular phenotype of colon epithelial cells in the in vitro derived colon organoids. Moreover, this unique research model enabled the study of the correlation of specific mutations to FAP manifestations in vivo in the donor parents.

## Materials and methods

### Ethics approvals

The use of spare in-vitro fertilization (IVF) embryos following preimplantation genetic diagnosis (PGD) for the derivation of hESC lines and for the study of genetic disease was approved by the Israeli National Ethics Committee (7/04-043) and was conducted in accordance with the guidelines of the Bioethics Advisory Committee of the Israel Academy of Sciences and Humanities. Clinical data analysis of the FAP families was performed under IRB 675/18.

### Cell lines

Three FAP-hESC lines were examined in this study: Lis25_FAP1 (FAP1), Lis30_FAP2 (FAP2)^25^, and Lis34_FAP3 (FAP3; NIH hESC line registry). All experiments were conducted using FAP-hESCs at passage 35–45. Two non-mutated APC hESC lines were used as controls: H9 and Hues13 (MTA with WiCell Inc.). We have previously reported full characterization of the FAP1 and FAP2-hESC lines^25^. The pluripotency of FAP3 was verified by analysis of the pluripotent stem cell nuclear marker OCT4 and the cell surface marker SSEA4 expression profiles (Suppl. Figure 1A). FAP3-hESCs also exhibited typical hESCs morphology and had a normal karyotype, as shown by chromosomal microarray analysis (CMA; Suppl. Figure 1B).

### Cell culture

hESCs were cultured on Geltrex-coated (ThermoFisher) plates in mTeSR1 medium (STEMCELL Technologies), supplemented with 100 µg/ml primocin (InvivoGen). hESCs were cultured in standard hESC medium (DMEM/F12, 20% KOSR (Invitrogen), 10 ng ml^−1^ basic fibroblast growth factor (FGF) (Peprotech), 1% MEM non-essential amino acids, 0.1 mM β-mercaptoethanol and 1 mM glutamine), were grown on a feeder layer of mouse embryonic fibroblast (MEF) cells. hESCs were passaged using accutase (Merck Biological Industries) and medium was supplemented with 10 µM ROCK inhibitor Y-27632 (Axon Medchem) for the first 24 h of culture, to inhibit apoptosis.

### Chromosomal microarray analysis

Genomic DNA was isolated from samples using the DNeasy Blood & Tissue Kit (Qiagen). The DNA was amplified, labeled, and hybridized to a 24sure V3 microarray (Illumina), according to the manufacturer’s protocol. Scanning was performed using an Agilent G2565CA scanner and the arrays were analyzed using the BlueFuse Multi software. The detected copy number variants (CNVs) were interpreted by referring to key public databases (ISCA, DGV, Ensembl, Decipher).

### Colonic epithelial differentiation

Differentiation of FAP- and WT-hESC lines into colon organoids was induced using a published protocol^27^. Briefly, to generate definitive endoderm, hPSCs were treated with 3 μM CHIR99021 (CHIR, Stem-RD) and 100 ng/ml activin A (R&D systems) in RPMI (Cellgro), for one day, and then with 100 ng/ml activin A in RPMI supplemented with 0.2% BSA (Gibco) for three days. These cells were than subjected to hindgut differentiation by treatment with 3 μM CHIR99021 and 500 ng/ml FGF4 (Peprotech) in RPMI supplemented with 1X B27 (Gibco) for four days. From day 8, cells were cultured in colonic medium comprised of advanced DMEM F12 (Invitrogen) supplemented with 1X B27 (Gibco), 3 μM CHIR99021, 300 nM LDN193189 (Axon) and 100 ng/mL EGF (R&D). The medium was refreshed every two days. On day 20, the cells were disaggregated to a single cell suspension and then re-suspended in Matrigel (BD Biosciences).

### RNA extraction and quantitative real-time PCR (qRT-PCR)

qRT-PCR and Western blot analysis were conducted as described previously^26^. Primer sequences are listed in Supplementary Table 1.

### Immunofluorescence

For hESC staining, cells were grown on MEF feeder cells in 24-well plates and fixed with 4% paraformaldehyde (PFA). For intra-cellular staining, cells were incubated in blocking solution (2.5% BSA in PBS) with 0.1% Triton, followed by incubation with a primary antibody diluted in blocking solution (1 h, room temperature), washed and then incubated with a secondary antibody for 1 h, and then counterstained with DAPI for nucleus localization. Bright-field phase and fluorescence images of cells were obtained using an Olympus IX51 inverted light microscope.

Whole organoids were stained by fixing them in 4% PFA, cryoprotecting them in 30% sucrose/PBS solution, embedding them in optimal cutting temperature (OCT) compound, and then snap-freezing them. Frozen samples were sectioned at 10 µm using a cryostat (Leica), and affixed to Superfrost Plus microscope slides. For intra-cellular staining, OCT-embedded sections were incubated in blocking solution (2.5% BSA in PBS) with 0.1% Triton and probed with fluorescently labeled antibodies. Primary antibodies and their dilution ratios are detailed in Supplementary Table 2.

### Immunohistochemistry

Samples were fixed in 4% PFA and embedded in paraffin. Immunohistochemistry was performed to detect the colon markers CDX2 and keratin 20, the stromal marker vimentin (VIM) and Ki67, a nuclear marker indicative of cellular proliferation. Staining was performed on a Ventana BenchMark Ultra Autostainer (Ventana Medical Systems Inc.). Analysis was performed on 10 random photos taken from each sample via light microscopy (20×). The VIM to Ki67/CDX2 ratios were determined using ImageJ software. Supplementry Fig. 2 outlines the quantification procedure performed to determine the proliferation rate in colon organoids. Primary antibodies and their dilution ratios are listed in Suppl. Table 3.

### DNA replication in proliferating cells

Cell cycle distribution was determined using the Click-iT EdU Alexa Fluor 647 Flow Cytometry Kit (Life Technologies), according to the manufacturer's protocol. Briefly, cells were pulse-labeled with 20 μM EdU for 2 h, at 37 °C, to label the cells in S phase, and then incubated with Click-iT EDU Alexa Fluor 647, for 30 min, at room temperature. Cells were then stained with FxCycle Violet (Invitrogen), for total DNA content, and analyzed using the BD FACSCanto II Flow Cytometer (BD Biosciences) and BD FACSDiva Software (BD Biosciences).

### RNA sequencing and bioinformatic analysis

Total RNA was extracted from hESCs (day 0) and on day 8 and day 20 of their differentiation into colon organoids, using the RNeasy Mini Kit (QIAGEN). Extraction was performed in two biological experiments. Library preparation and RNA sequencing were performed on Illumina NovaSeq6000, at a commercial laboratory (Macrogen Inc., South Korea). Raw sequencing data was trimmed and filtered using fastp 0.19.6^28^, then aligned to the GRCh38 assembly using STAR 2.7.1a^29^. Differential expression analysis was performed using DESeq2 1.24.0^30^ and gene set enrichment was performed using clusterProfiler 3.16.0^31^, both on R version 3.6.1. A heatmap was created using ComplexHeatmap 2.4.2^32^. Full details of the RNA-seq data can be found in https://drive.google.com/drive/folders/1nj1frTfSNOAyuEsF9BMRPcuVviRQtzmy?usp=sharing.

### Statistical analysis

For all experiments, three independent experiments were carried out unless otherwise stated. p values were calculated using the unpaired two-tailed Student’s t-test and ANOVA, both computed with SPSS, and are represented as; *p < 0.05, **p < 0.01, ***p < 0.001, and ****p < 0.0001.

## Results

### APC germline mutation affects FAP-hESC differentiation into colon organoids

**Figure 1:**
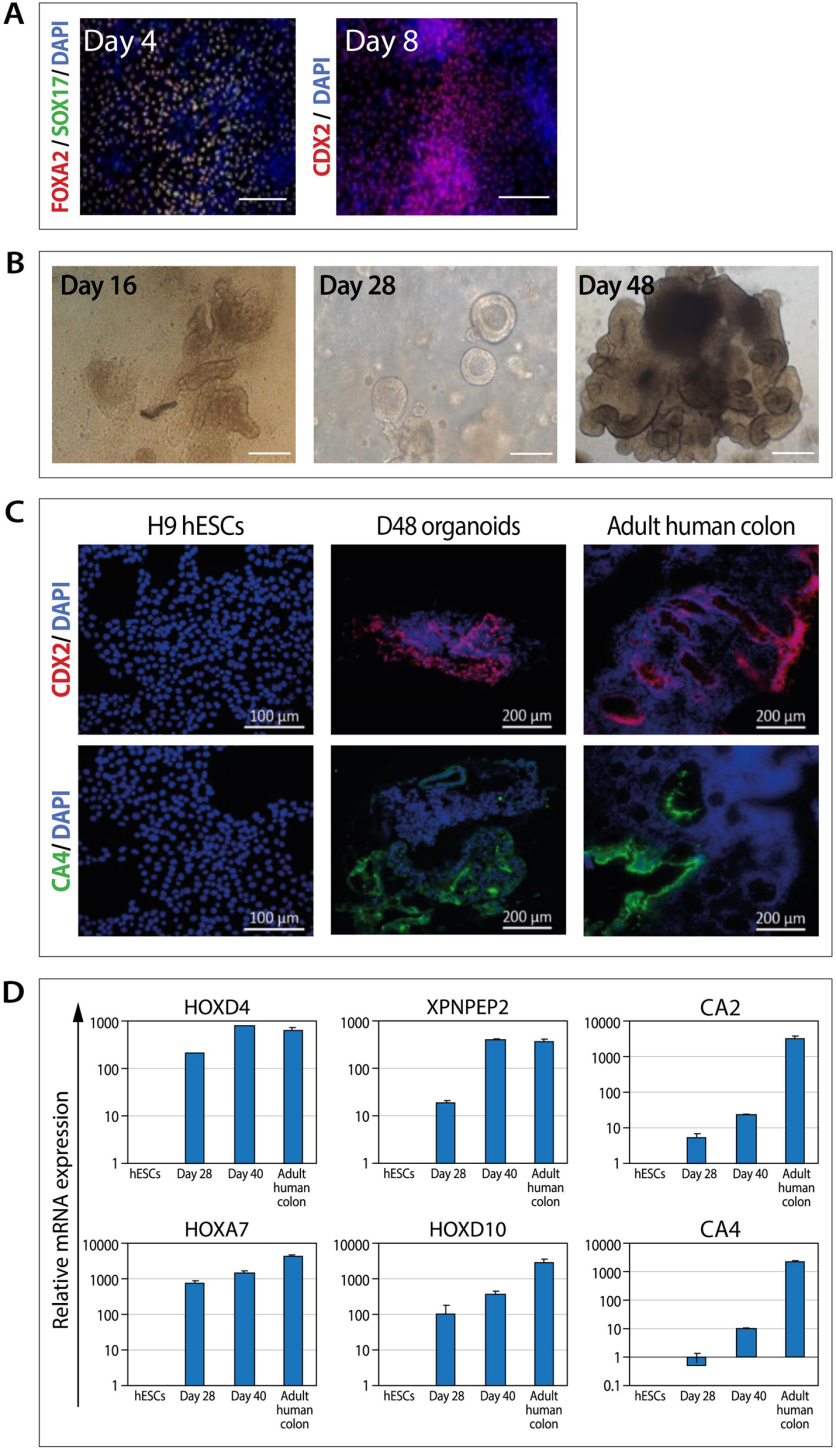
Characterization of hESC-derived colon organoids. (**A**) Immunostaining of H9 hESC-derived definitive endoderm (day 4) and hindgut endoderm (day 8). Scale bars: 200 µM. (**B**) Representative bright-field images of developing hESC-derived colon organoids. Scale bars: 200 µM. (**C**) Immunostaining of hESC-derived colon organoids. Adult human colon tissue samples and H9 hESCs served as controls. Scale bars: 200 µM. (**D**) RT-PCR analysis of colonic cell markers in hESC-derived D28 colonic spheroids and D40 colon organoids. Adult human colon tissue samples and H9 hESCs served as positive and negative controls, respectively. Data are presented as relative mean expression (± standard deviation) of triplicate samples from one experiment.

In order to mimic the natural niche of CRC development, hESCs were differentiated into colon organoids. Differentiation of control hESCs (H9) into colon organoids was induced using a recently published protocol^27^. Generation of definitive endoderm (day 4) and hindgut endoderm (day 8) was confirmed by the expression of the epithelial markers FOXA2, SOX17 and the intestinal epithelial marker CDX2 (Fig. 1A). Cells were then cultured to generate colonic epithelial cell aggregates (Fig. 1B, day 16). By day 20, cells were dissociated and embedded in Matrigel where they continued to develop into 3D cell clusters with spheroid morphology (Fig. 1B, day 28), which then turned into complex and mature colon organoids (Fig. 1B, day 48). The day-48 colon organoids expressed the colonic markers CDX2 and CA4, similarly to the adult human colon sample that served as a positive control (Fig. 1C). In order to further confirm their colonic phenotype, qRT-PCR analysis demonstrated that colon spheroids (day 20) and organoids (day 40) expressed relatively high levels of six colonic markers, as compared to their undetected levels in the pluripotent, undifferentiated, hESCs (Fig. 1D).

**Figure 2:**
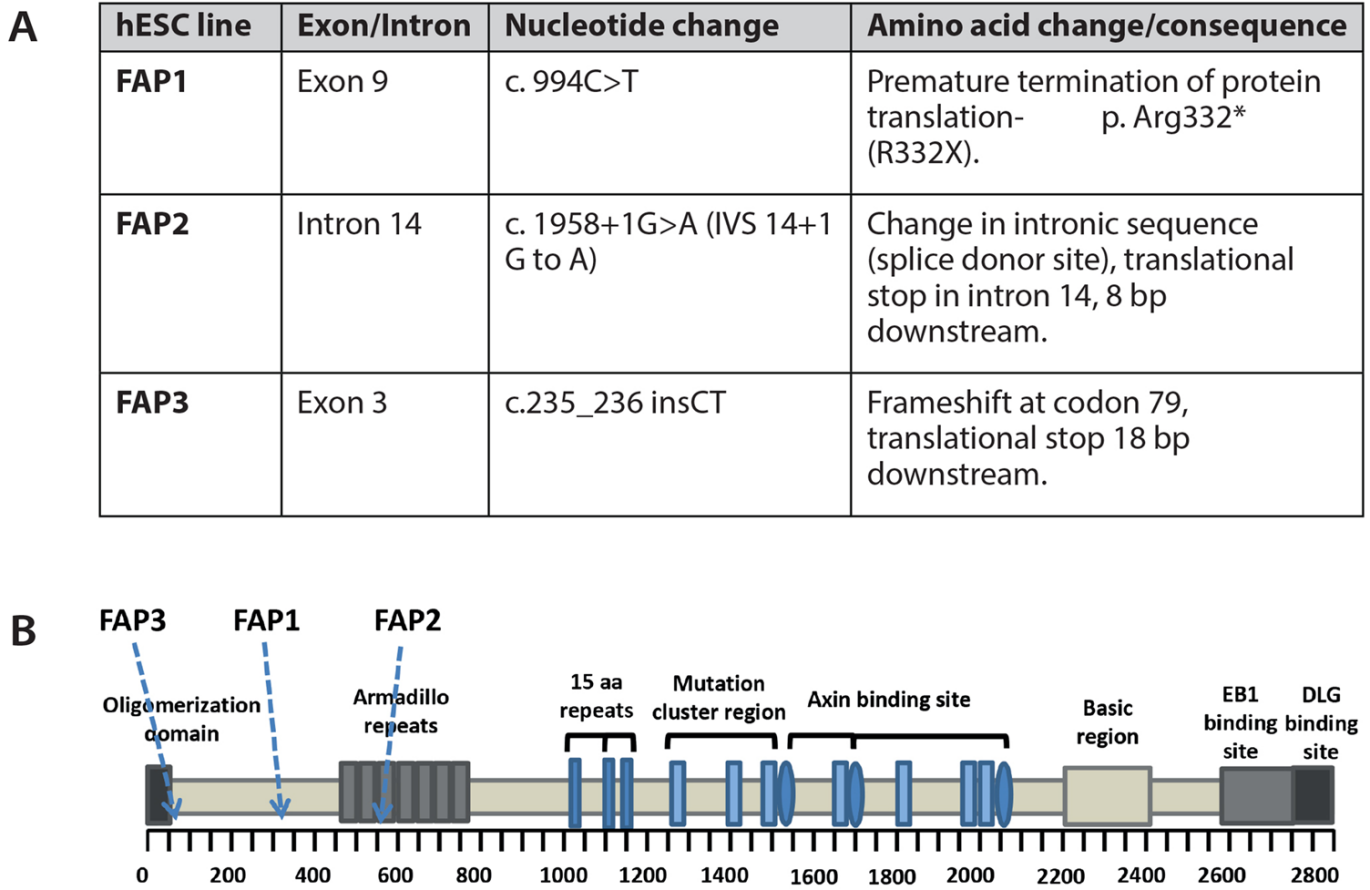
A molecular summary of APC germline mutations in the FAP-hESC lines. (**A**) Description of the type and location of APC germline mutations in each of the three FAP-hESC lines derived following preimplantation genetic diagnosis (PGD). (**B**) Schematic structure of the APC protein. Positions of the APC germline mutations in FAP1, FAP2 and FAP3-hESC lines are indicated by arrows. O-oligomerization domain, MCR- mutation cluster region, basic microtubule binding site.

**Figure 3:**
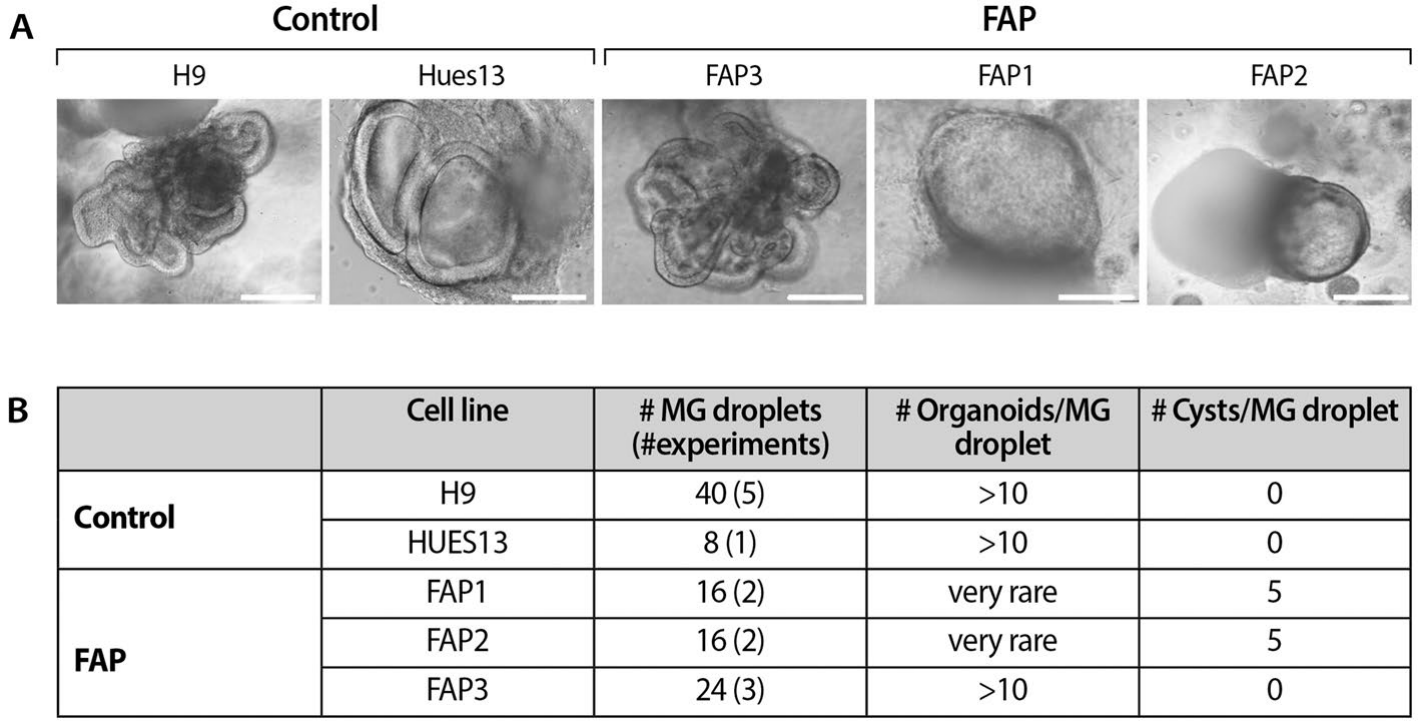
Capability of FAP-hESC lines to form colon organoids. (**A**) Representative images of 3D colon organoids derived from control hESC lines (H9 and Hues13), and FAP-hESC lines (FAP 1–3), each bearing a different germline APC mutation. Scale bar: 200 µm. (**B**) Number and complexity of day-48 colon organoids of control (H9 and Hues13) and FAP-hESC lines (FAP 1–3). Presented are the number (#) of experiments, # of Matrigel (MG) droplets created in all experiments performed, average # of organoids developed/MG droplet and average # cysts/MG droplet.

To investigate the impact of the heterozygous APC germline mutation on the differentiation potential of hESCs into colon organoids, three FAP-hESC lines derived from three different families carrying various APC germline mutations (Fig. 2), were utilized. FAP1 and FAP2 hESC lines carry a germline mutation that produce a truncated protein from the mutated allele; in FAP1-hESCs, there is a stop codon in amino acid 332 (exon 9) on the APC gene, while in FAP2, the mutation is in the splice site of intron 14, introducing a premature termination codon. In FAP3, an insertion of two nucleotides (CT) in position 235 (exon 3) of the APC mRNA sequence, leads to a frameshift mutation in the 5′ end of the *APC* gene, resulting in lack of expression of the mutated allele. These three FAP were differentiated into colonic organoids and exhibited a diverse potential (Fig. 3A,B). While FAP3 was able to reproducibly self-organize into complex three-dimensional colonic structures (> 10 organoids/Matrigel droplet), similar to those of the two WT-hESC lines (H9 and Hues13 > 10 organoids/Matrigel droplet), FAP1 and FAP2-hESCs grew in an unorganized manner and developed only cyst-like structures and cell aggregates of various shape, occasionally with luminal part (4–5 cysts/Matrigel droplet). In summary, the inability of FAP1 and FAP2 to generate colon organoids is conclusive as we have repeated these differentiation experiments 13 times and a single complexed organoid was generated only in one droplet in one experiment (i.e. a very rare event), from either FAP1 or FAP2 lines, while the positive control (WT) as well as FAP3 line generated complexed colon organoids in all experiments.

**Figure 4:**
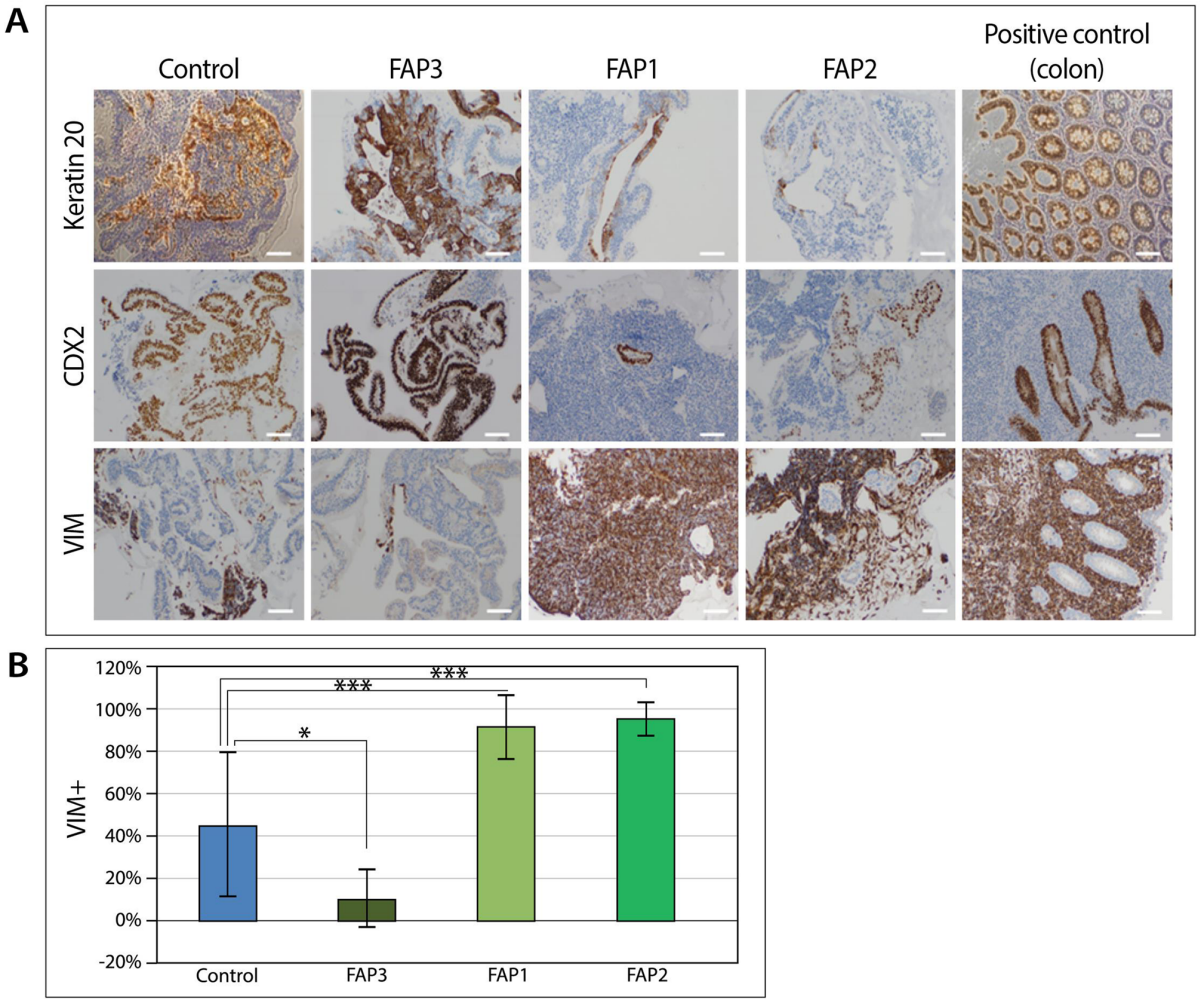
Lineage characterization of cells within colon organoids. (**A**) Representative images of immunohistochemical staining of colon epithelial markers keratin 20 and CDX2 and the mesodermal marker VIM in control and FAP-colon organoids on day 48 of differentiation. Normal colon tissue served as a positive control. Scale bars: 200 µM. (**B**) Quantification of VIM^+^ cells by the ImageJ image processing program. Analyses were performed on 10 photos randomly selected from each sample (*1*0 ×). *p < 0 .05, ***p < 0 .005; One-way ANOVA.

Day-48 control and FAP3-hESCs-derived organoids demonstrated higher expression levels of the colon epithelial markers CK20 and CDX2 compared to FAP1 and FAP2-organoids (Fig. 4A), indicating that they are predominantly composed of epithelial cells. Accordingly, vimentin (VIM) a mesenchymal protein, was highly expressed in FAP1 and FAP2 derived structures and was expressed at lower levels in the more developed organoids derived from FAP3 and control-hESCs (Fig. 4B). This same analysis revealed a dominant component of supportive mesodermal tissue in FAP1 and FAP2 (91.7% and 95.4%, respectively) as compared to WT and FAP3-derived colon organoids (44.9% and 10.6%, respectively; Fig. 4B). Taken together, FAP1 and FAP2 hESCs present impaired potential to form colon organoids as compared to FAP3, which generated complex colon organoids similar to those of the control.

### The effect of APC germline mutations on cell proliferation

**Figure 5:**
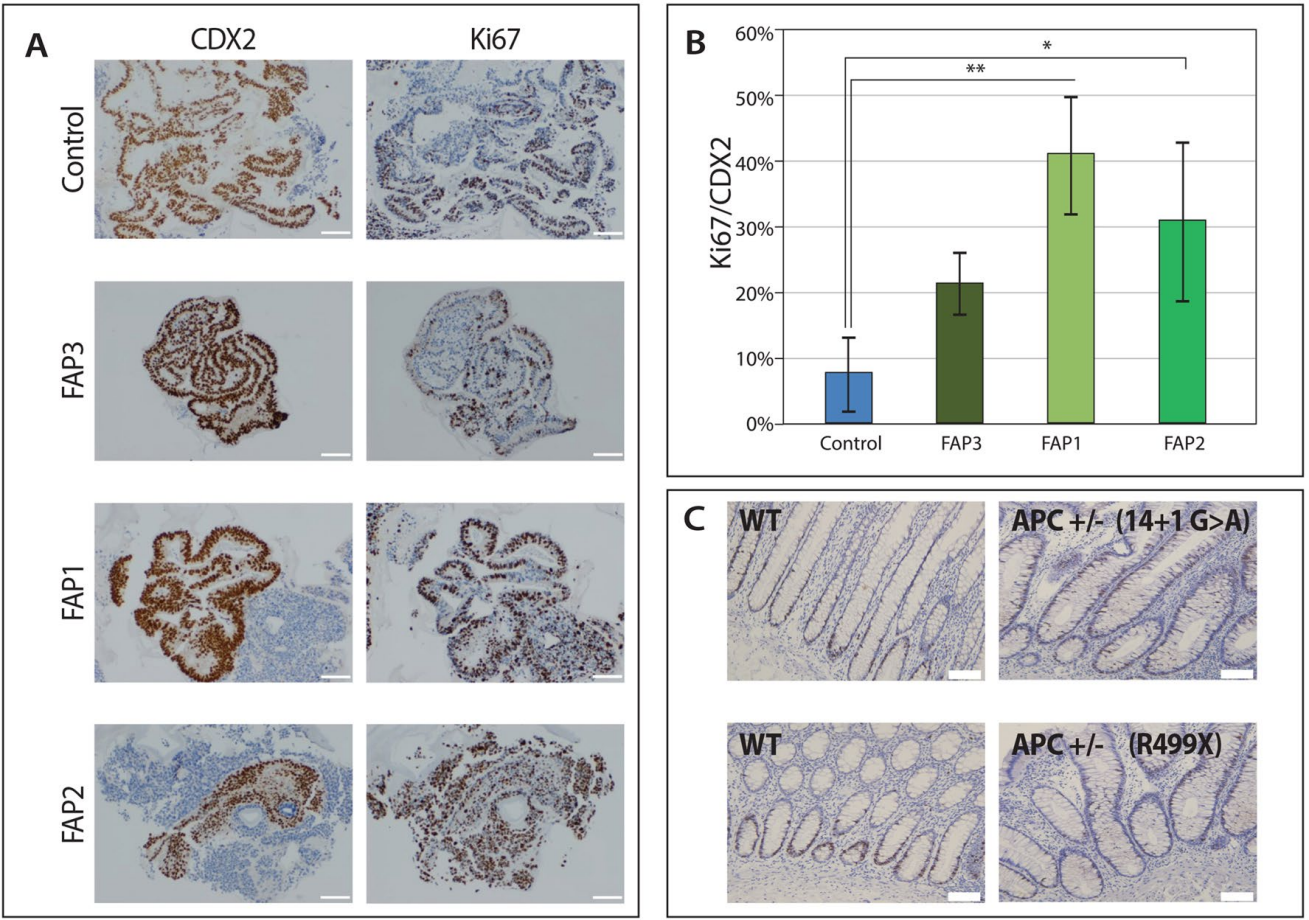
Cell proliferation in FAP-hESCs and their derived colon organoids. (**A**) Representative photos of immunohistochemical staining of the cell proliferation marker Ki67 (right) and the colon epithelial marker CDX2 (left) in control and FAP-colon organoids on day 48 of differentiation. Scale bars: 100 µM. (**B**) Quantification of Ki67/CDX2-positive cells was performed using the image processing program ImageJ, using ImmunoRatio software, on 5 different fields taken from each sample (magnification: 10 ×). One-way ANOVA *p < 0.05, **p < 0.01. (**C**) Representative images of the Ki67 immunostaining pattern in normal control colonic mucosa (WT) versus normal-appearing crypts from FAP patients (APC + / −). Scale bars = 100 µM.

Embryos at early development and ESCs are characterized by rapid cell proliferation; as cells differentiate, the proliferation rate generally decreases. To assess the effect of the heterozygous APC mutations on hESC proliferation, the percentage of pluripotent hESCs in the S-phase of the cell cycle was determined. Similar proliferation rates were observed for all undifferentiated pluripotent hESCs (both WT-control and the three FAP-hESC lines; Suppl. Figure 3). In contrast, differentiated FAP1 and FAP2 colon organoids contained a significantly higher fraction of proliferating colon epithelial cells (40% and 31%, respectively) as compared to WT (7%; *p* < 0.05) and FAP3 colon epithelial cells (Suppl. Figure 1; Fig. 5A,B). These findings were in accordance with their potential to form colon organoids, with complexed structure (FAP3 and WT-control) showing a lower proliferation rate, than the simple organoids (FAP1 and FAP2).

To determine whether these in vitro findings represent the effect of APC germline mutations on cell proliferation in vivo, Ki67 expression in normal-appearing crypts derived from FAP patients was measured. It is well established that in the normal colonic epithelium, the proliferative cells reside within the bottom region of the crypt, comprising the crypt stem cells that are also the cells-of-origin of intestinal cancer^33–35^. As expected, Ki67 expression in normal control colonic mucosa (non-FAP patients) was observed mainly in the lower crypt (Fig. 5C; WT). Interestingly, in seemingly non-pathological (non-adenomatous) crypts of FAP patients (Fig. 5C; APC+/−), Ki67-positive cells were abnormally and randomly distributed all along the crypt, even extending towards the crypt surface. These results demonstrate that the in vitro-derived colon organoids indeed reflect the abnormal disease phenotype and serve as a good research model for early colon cancer, in general, and for FAP mutations, in particular.

### Transcriptome analysis of FAP colon organoids

**Figure 6:**
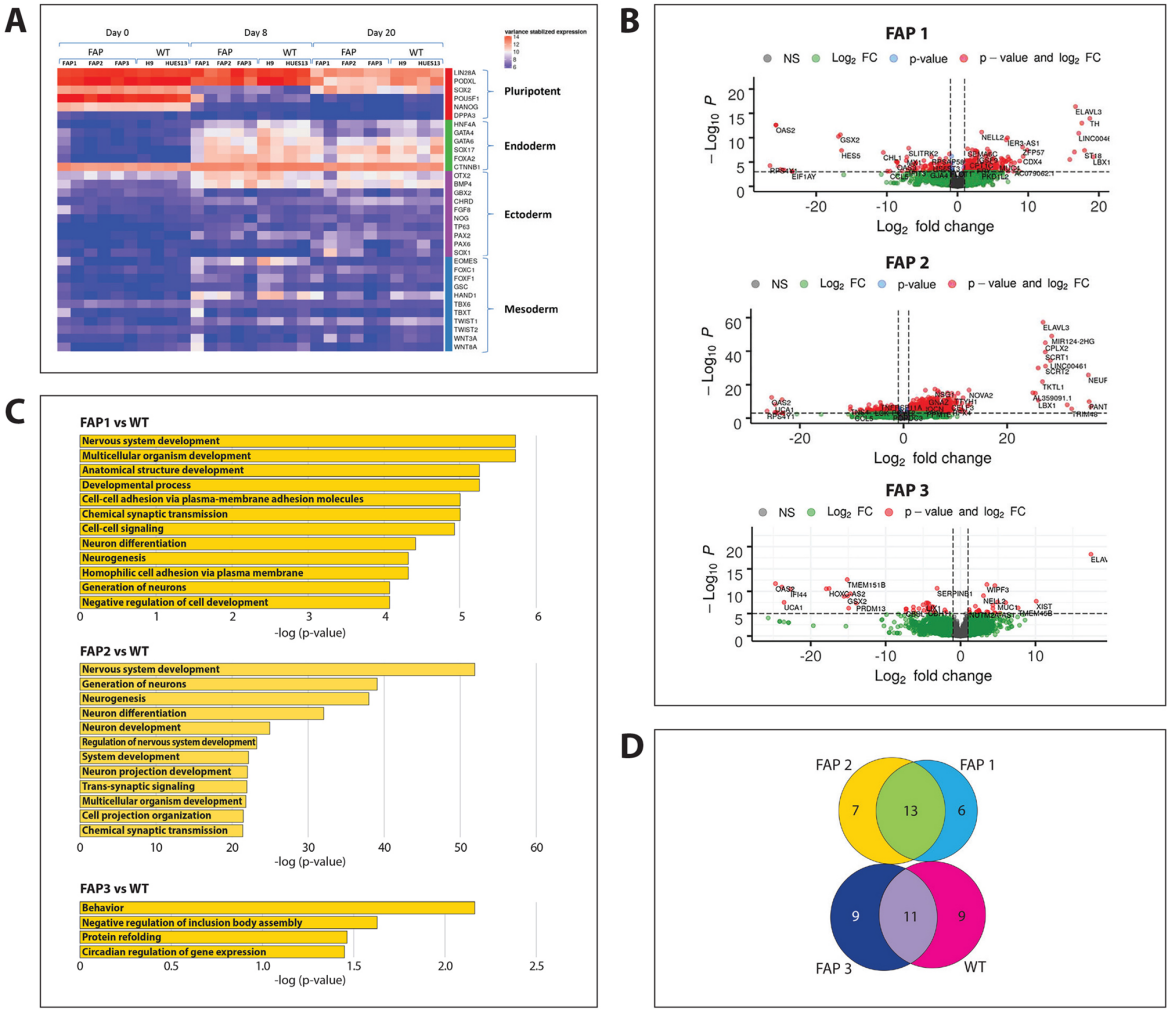
Transcriptome of FAP-hESC lines at different stages of differentiation into colon organoids. (**A**) Heat map of differentially expressed genes (DEGs) during differentiation of FAP-hESCs into colon organoids. Columns represent samples and rows represent genes, categorized in relation to pluripotency and to cell lineage (endoderm, ectoderm, and mesoderm). The heat map illustrates lower (blue) to high (red) gene expression levels with distinct transcriptional profiles across the differentiation process. (**B**) The volcano plots of DEGs in all three FAP-hESC lines compared to WT, on day 8 of differentiation into colon organoids. (**C**) Gene ontology biological process analysis of upregulated DEGs in FAP compared to WT cells, on day 8 of colon organoid differentiation. (**D**) Venn diagram showing the overlap of biological processes at day 8 of differentiation in FAP and WT cells.

To determine which molecular changes are affected by the various heterozygous APC mutations leading to tumorigenic transformation of the cells long before any clinical change is observed, RNA sequencing was performed at different time points during differentiation of hESCs into colon organoids. The heat map of differentially expressed genes (DEGs) showed high expression of pluripotency genes in all three FAP-hESC lines (day 0), as well as in the control WT lines, demonstrating that the germline APC mutation does not affect pluripotency at the cellular or gene expression levels (Fig. 6A). During differentiation into colon organoids, the expression of these genes gradually decreased (day 8 and 20), which was paralleled by a gradual increase in endodermal marker expression, while the expression of ectodermal and mesodermal markers was mostly unchanged (Fig. 6A). Most changes in DEGs between FAP and WT cells were already observed by day 8 of differentiation, prior to any morphologically apparent differences arose between them. Volcano plots of day-8 expression data demonstrated a massive gene upregulation in FAP1 and FAP2 organoids (66% and 74%, respectively, of all genes; Fig. 6B) compared to WT, while in FAP3 organoids, 55% of the genes were upregulated compared to WT. Gene-Ontology (GO) annotation of these upregulated DEGs (Fig. 6C) showed upregulation of neurogenesis-related processes unrelated to colon formation in FAP1 and FAP2 organoids but not in the more mature FAP3 colon organoids. These findings were supported by pathway analysis that identified glutamate metabolism enrichment in day-8 FAP1 and FAP2 colon organoids (Suppl. Figure 5). Most importantly, Wnt signaling, which is the main pathway directly antagonized by APC, was significantly upregulated in FAP1 & FAP2 cells (Supp. Figure 5), but not in FAP3. In fact, no enrichment signaling pathways were observed in FAP3 as compared to WT. On day 20 however, differences in DEGs among FAP1, 2 and 3 were not pronounced (Suppl. Figure 4). Moreover, GO analysis of DEGs upregulated between day 0 and day 8, demonstrated that 11 biological processes were shared between FAP3 and WT cells, while not a single biological process was shared between FAP1/FAP2 and WT (Fig. 6D). Furthermore, while FAP3 and WT shared processes such as protein and Golgi movement, which are typical to colon absorption and its normal function, FAP1 and FAP2 exhibited more immature processes, characteristic of early development (Suppl. Table 4). Taken together, already on day 8 of in vitro differentiation into colon organoids, FAP1 and FAP2 cells failed to upregulate biological processes that are critical for proper colon differentiation.

### Phenotype-genotype correlation between FAP embryo donors and the genetically related in vitro-derived colon organoids

The variable differentiation capacity among the different FAP-hESCs-derived colon organoids carrying only the germline mutation, suggested a possible role of the location and type of the APC germline mutation and predisposition to tumorigenic transformation. Clinical phenotype and management data of the FAP-affected patient donors is summarized in Table 1. Patients harboring the FAP1 and FAP2 APC mutations presented a more severe clinical manifestations of the disease, with higher polyp burden and had undergone colon removal surgery due to increased risk for developing CRC. The patient harboring the FAP3 APC mutation was diagnosed with attenuated FAP, and carried only several adenomatous polyps which were removed during the colonoscopy procedure and did not necessitate any further surgical intervention. Taken together, these results suggest that the type of APC germline mutation determines the clinical phenotypic severity of FAP and may reflects the ability to generate colon organoids in vitro.

**Table 1:** Clinical characteristics and management of FAP affected parents who donated their embryos for the derivation of FAP-hESC lines.

hESC line	1st colonoscopy			Colorectal surgey			Extra colon manifestations		
	Reason for 1st colonoscopy	Age	Berden of polyps	Surgery	Polyps after surgery	Desmoid	Duodenal adenoma	Gastric polyps	Papillary thyroid carcinoma
FAP1	Rectal bleeding	40	50	Ileal [ouch-anal anastomosis (Age 40)	No polyps	No	No	No	No
FAP2	No symptoms, family history of FAP	50	50	Subtotal colectomy with rectal stump (Age 14)	Polyps in rectal stump	No	No	Carpet of small fundic gland polyps	No
FAP3	No symptoms, family history of FAP	27	3	No	–	No	No	No	No

## Discussion

The current understanding of CRC stems either from the examination of patient samples and cell lines, which are highly variable and provide only a post hoc view of disease progression, or from investigation of animal models, which often fall short in faithfully recapitulating the human condition^19–24^. hESCs carrying specific mutations characterizing human genetic disorders provide a valuable tool for studying the pathophysiology of these diseases in humans^36,37^. The advantages of these cells include their normal karyotype, rapid growth and self-renewing capacity and their broad differentiation potential. In efforts to establish a clinically relevant model to study the role of heterozygous APC germline mutations in initiating tumorigenic transformation, our lab derived several hESC lines from FAP blastocysts, carrying different APC germline mutations^25^. These FAP-hESC lines were subsequently induced to form 3D colon organoids, using the technology recently developed by Crespo et al^27^. The three different FAP-hESC lines exhibited variable capacity to form colon organoids, which correlated with the severity of the FAP disease in the affected embryo donor parents. Similar effect of a diseased mutation on the altered morphology of the derived organoids was shown also when using adult stem cells rather than embryonic stem cells to model breast and ovarian tumor^38,39^. However, FAP-iPSCs carrying different heterozygous APC mutations successfully formed intestinal or colon organoids, regardless the type or the location of the APC germline mutation^27,40^. This feature of the organoids derived from the mutated stem cells to represent the disease-driving mutation, differs among studies, may be due to the differences between hESCs and iPSCs or due to their different genetic background. While different genetic background among hESC lines has no effect on their pluripotent state as we can infer from the transcriptome analysis at day 0, we cannot rule out the possibility that phenotypic differences between lines during differentiation to colon organoids can be attributed to their different genetic background.

It has been shown that the location of germline mutations in the *APC* gene is the most striking source of variability affecting the number of polyps that will develop in the colon of FAP patients^13,41,42^. In accordance, the current study found a correlation between the severity of the disease in FAP patients who donated their affected embryos for our FAP-hESC lines and the 3D structure of their derived colon organoids and the location of their germline APC mutations. It was previously suggested that a stable truncated APC protein may act in a dominant negative fashion to inactivate APC transcribed from the WT allele^43^. Homodimerization of APC at the amino-terminus implies a possible dominant negative mode of action for mutant APC in heterozygous cells, in which shorter proteins can functionally inactivate the full-length WT protein^44,45^. The first 170 amino acids are sufficient for APC homodimerization in-vitro, an association which requires the 45 amino acids only^43^. FAP3 carries a heterozygote frameshift mutation that is predicted to be in amino acid 60, presumably resulting in lack of function of the mutated allele (‘first hit’), but the normal allele express the APC since the ‘second hit’ didn’t occur yet. Consequently, in the absence of a gain-of-function effect, the patient presents only mild disease, and the corresponding complexed organoids derived in vitro are similar to those of WT hESCs expressing the two normal alleles of the APC. Therefore, we hypothesis that while the APC germline mutations in FAP1 and FAP2 may result in a truncated protein that can cause a dominant-negative effect on the normal allele, the germline mutated protein translated in FAP3 is likely too short to interfere with the normal APC protein.

In order to further understand the differences in the differentiation potential of the FAP-hESC lines and their derived colon organoids and to identify genes whose expression is altered as a direct result of the loss of a single APC allele, RNA sequencing was performed at different time points of the differentiation process. These data demonstrated a significant gene expression alteration already on day 8 of differentiation, which directed the cells to either colon epithelial lineage (in control WT and FAP3), or towards the non-colonic neurogenic lineage (in FAP1 and FAP2). Upregulation of neurogenic genes was also reported in prostate cancer^46^, head and neck cancer^47^, pancreatic cancer^48^ and colon cancer^49,50^. In mouse intestinal organoids, Farin et al^51^ demonstrated that increased Wnt-activity changed the growth pattern of the organoids from branched to cyst-like, as observed here in the different human FAP organoids. Moreover, aberrant activation of Wnt signaling is frequently observed in human cancers, and is considered to be a regulator of CRC development^52^. Activation of the Wnt pathway and the enhanced proliferation capacity in FAP1 and FAP2 organoids comprise the very early stages activated already by the 'first hit' in FAP patients, suggesting that the heterozygous FAP1 and FAP2 mutations lead to premature colon development as compared with FAP3.

Differentiation into colon organoids decreased proliferation in the normally developed FAP3 and WT organoids. In contrast, FAP1- and FAP2-derived colon epithelial cells showed significantly increased proliferation, which correlated with their relatively reduced differentiation. These observations aligned with the reported increased cell proliferation in human colon organoids derived from FAP patient-iPSCs and with their predisposition to adenoma formation^27^.

In FAP patients, colon polyposis develops only after occurrence of an additional somatic genetic mutation in the WT APC allele, consistent with the ‘two hit’ hypothesis relating to cancers arising from defective tumor suppressor genes^11^. The notion that a heterozygous APC mutation (‘one hit’) might be sufficient to initiate tumorigenic transformation was suggested years ago by Kopelovich et al^53,54^ who observed profound genetic alterations in skin fibroblasts derived from FAP patients. Other studies reported genetic and proteomic alterations in morphologically normal colon crypts of FAP patients^55,56^. Moreover, a recent single-cell RNAseq analysis of normal mucosa of patients with FAP demonstrated that cells from normal colon epithelium already exhibit enhanced metabolic processes and proliferative activity compared with cells from the normal colon epithelium of patients with sporadic CRC^57^. In addition, in the normal colonic epithelium, the proliferative cells are localized to the bottom of the crypt, comprising the cells-of-origin of intestinal cancer^33–35^. Interestingly, our results show that the ‘first hit’ in APC is sufficient to molecularly alter the cells resulting in proliferating cells that are randomly distributed all along the crypt, as was shown also by Boman et al^58^. These results support our proposal that pathogenic events in the normal epithelium may occur long before any clinical manifestation. The presented results are the first to use FAP-hESCs to provide key data demonstrating that a heterozygous APC mutation is sufficient to cause molecular defects that impact basic cellular functions, such as differentiation and proliferation. These alterations likely represent the earliest molecular changes occurring during colon carcinogenesis.

In conclusion, this study suggest that FAP-hESC-derived colon organoids may serve as an informative tool to predict the clinical severity of FAP, based on APC mutation analysis, as well as a good model to study colonic carcinogenesis. Furthermore, it shows that a single heterozygous APC mutation can alter the molecular and cellular phenotype of cells and provide a selective advantage of adenoma formation during CRC development. The presented findings support the notion that the colon of FAP patients is predisposed to cancer long before clinical manifestation. However, as the number of available FAP-hESCs in this study is limited further study on more FAP-hESC lines is essential to reinforce our findings. Moreover, further analysis of the colonic progeny of FAP-hESCs will likely advance the identification of new target genes whose expression is altered directly as a result of the loss of a single APC allele. These findings will hopefully enhance our understanding of the earliest events leading to CRC development and to the design of specific treatments that can prevent the acquisition of the 'second hit', and to escape the transition to adenomas and further progression to carcinoma. This work also demonstrated the utility of stem cell-derived colon organoids in exploring the pathophysiology of genetic disorders in a clinically relevant environment that can also serve for screening of potential drug.

## Supplementary Information

Supplementary Information
