# Supplementary material for: Heterozygous APC germline mutations impart predisposition to colorectal cancer
Source: Sci Rep. 2021 Mar 4;11:5113. doi: 10.1038/s41598-021-84564-4 (PMC7933349; doi:10.1038/s41598-021-84564-4)
Supplement: Supplementary file 1 — Supplementary Information [file 41598_2021_84564_MOESM1_ESM.docx]

**Supplementary figures and tables Legends**

**Suppl. Fig. 1.** Pluripotency characterization and chromosomal microarray analysis of the FAP3-hESC line.

**
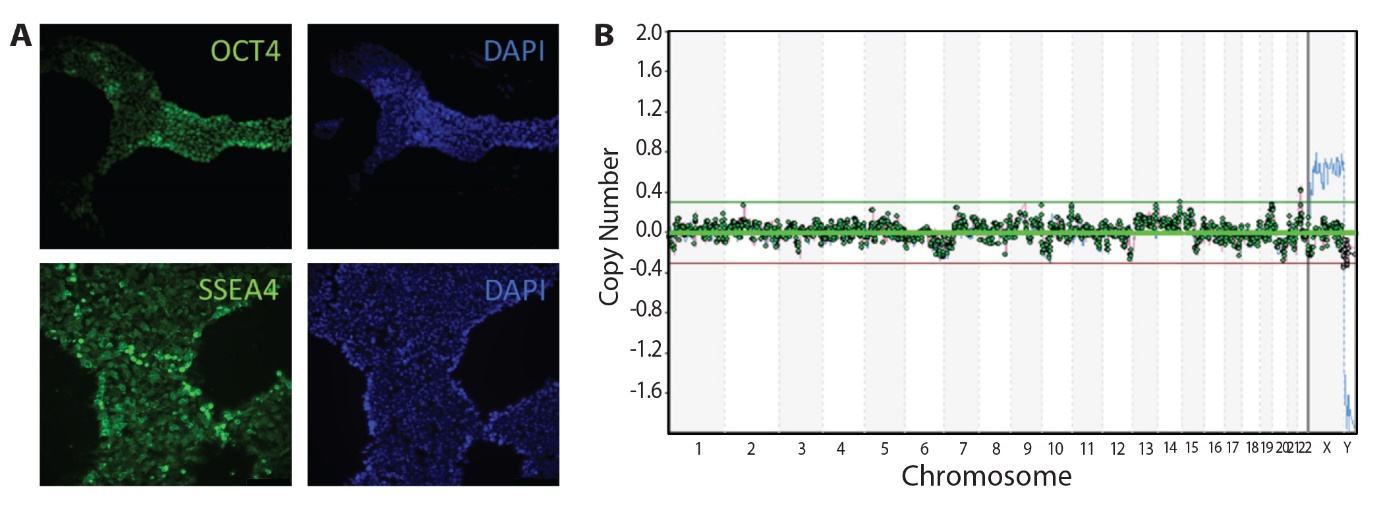
**

**(A)** Immunostaining for two pluripotent markers, OCT4 (green) and the cell surface marker stage-specific embryonic antigen-4 (SSEA4; green). The nuclear marker DAPI is stained in blue. Scale bars: 100 µM. **(B)** Chromosomal microarray analysis (CMA) of copy number variations in FAP3-hESC DNA. The plots show the relative copy numbers (y axis) of each chromosome (x axis), demonstrating a normal karyotype, with no duplications or deletions. Green and red lines represent thresholds for normal karyotype.

**Suppl. Fig.2.** The quantification procedure performed to determine the proliferation


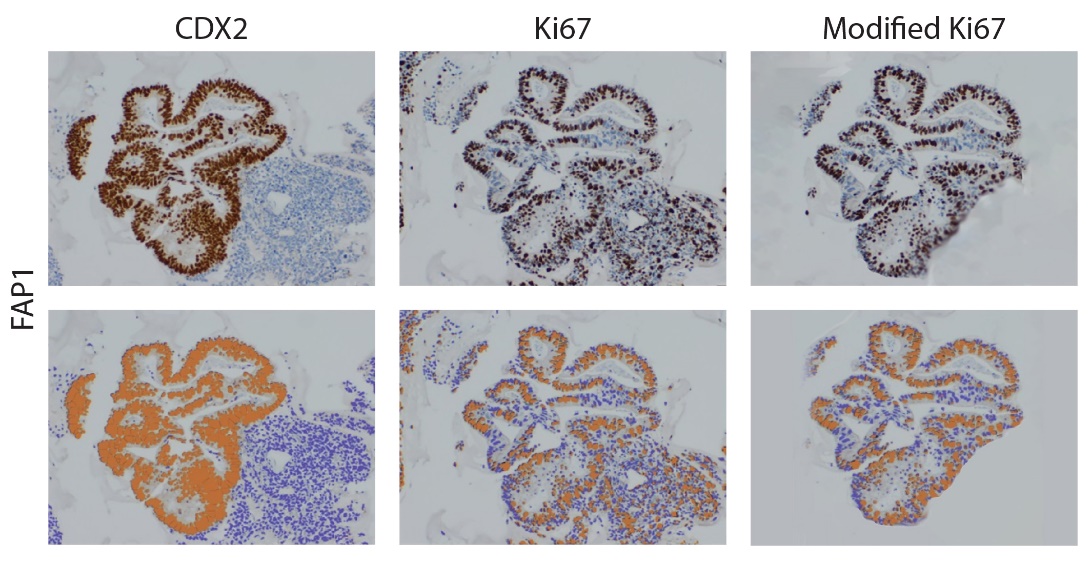


Only CDX2+ cells were analyzed for Ki67 co-staining.

**Suppl. Fig.3 –** Proliferation rate of the pluripotent hESC lines

**
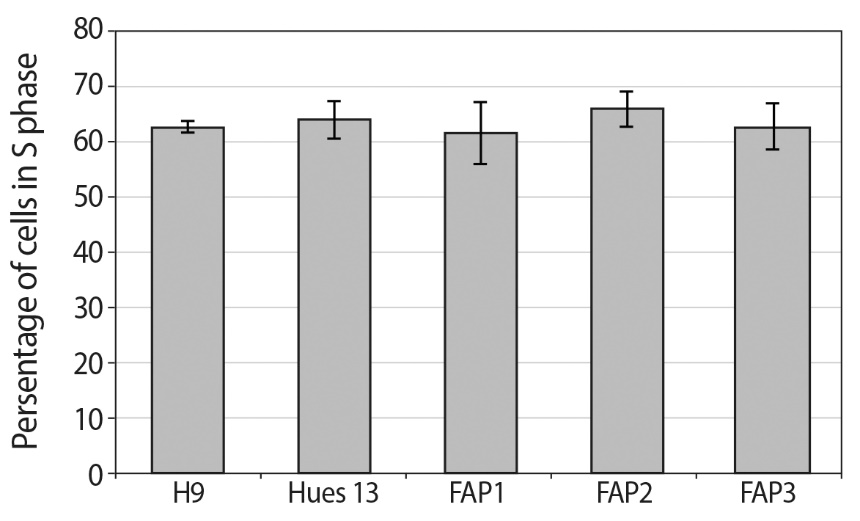
**

A histogram showing the percentage of cells in the S-phase of the cell cycle, as determined by DNA staining with FxCycle Violet, followed by FACS analysis.

**Suppl. Fig.4** - Volcano plots of differentially expressed genes in all three FAP-hESC lines: **(A)** FAP1, **(B)** FAP2, **(C)** FAP3, compared to WT, on day 20 of colonic differentiation.

**
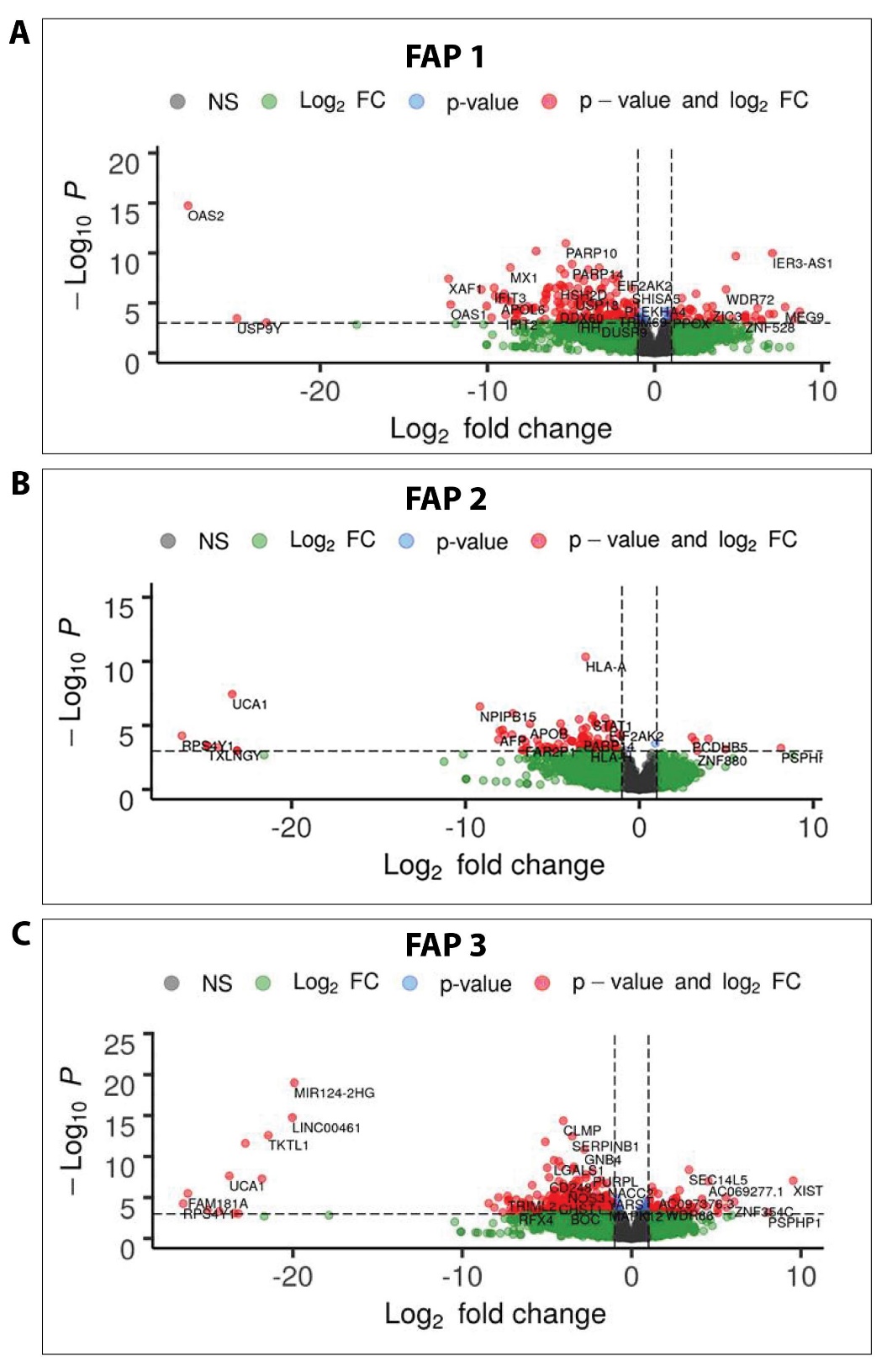
**

**Suppl. Fig. 5 -** (corresponding to Fig.5) - Gene ontology pathway processes analysis of upregulated differentially expressed genes in FAP compared to WT cells, on day 8 of colonic differentiation.


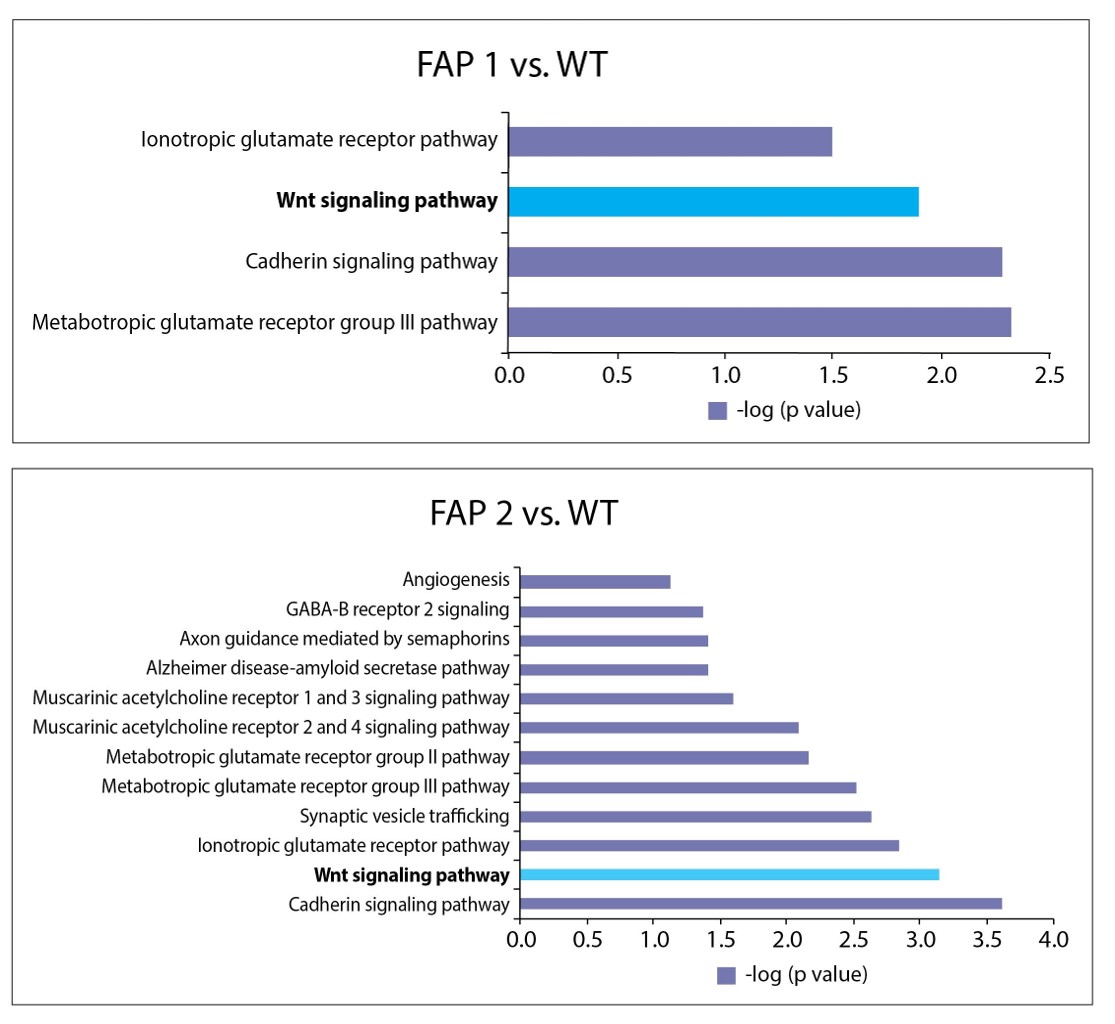


**Suppl. Table 1. Real-time PCR primers for analysis of transcript levels**

| **Gene** | **Forward** | **Reverse** |
| --- | --- | --- |
| GAPDH | CTCCTGCACCACCAACTGCT | GGGCCATCCACAGTCTTCTG |
| HOXD4 | CTACCCCTGGATGAAGAAGG | AGTTCTAGGACTTGCTGCCG |
| HOXA7 | CAATTTCCGCATCTACCCCT | GGAACTCCTTCTCCAGCTCC |
| CAII | TGATAAAGCTGCGTCCAAGA | GTCACTGAGGGGTCCTCCTT |
| HOXD10 | GTGCAGGAGAAGGAAAGCAAAG | TAACGCTCTTACTGATCTCTAGGC |
| CA4 | TCAGAGGACTCTTTGCTGTCC | AACCTCGTAGCACCAGTGTG |
| XPNPEP2 | GTGGCTCTCGTGGTAGAAGC | CGATGAGGTTCCGGTCATAG |

**Suppl. Table 2. List of primary antibodies used for immunostaining**

| **Antigen** | **Species** | **Vendor** | **Catalog Number** | **Dilution** |
| --- | --- | --- | --- | --- |
| OCT4 | mouse | Santa-Cruz | sc-5279 | 1:60 |
| SSEA4 | mouse | Cell signaling technology | CST-4755S | 1:200 |
| CDX2 | rabbit | Abcam | ab76541 | 1:500 |
| CA4 | mouse | R&D Systems | MAB2186 | 1:25 |
| FOXA2 | rabbit | Abcam | ab108422 | 1:500 |
| SOX17 | mouse | Abcam | ab84990 | 1:500 |

**Suppl. Table 3. List of primary antibodies used for immunohistochemistry**

| **Antigen** | **Species** | **Vendor** | **Catalog Number** | **Dilution** |
| --- | --- | --- | --- | --- |
| CK20 | mouse | Cell-Marque | 320M-16 | 1:100 |
| CDX2 | rabbit | Cell-Marque | 235R-16 | 1:100 |
| Ki67 | rabbit | Cell-Marque | 275R-16 | 1:200 |
| VIM | mouse | Dako | M0725 | 1:100 |

**Suppl. Table 4** (corresponding Fig.6C) **- List of overlapping biological processes activated on day 8 of differentiation in FAP and WT cells**

**
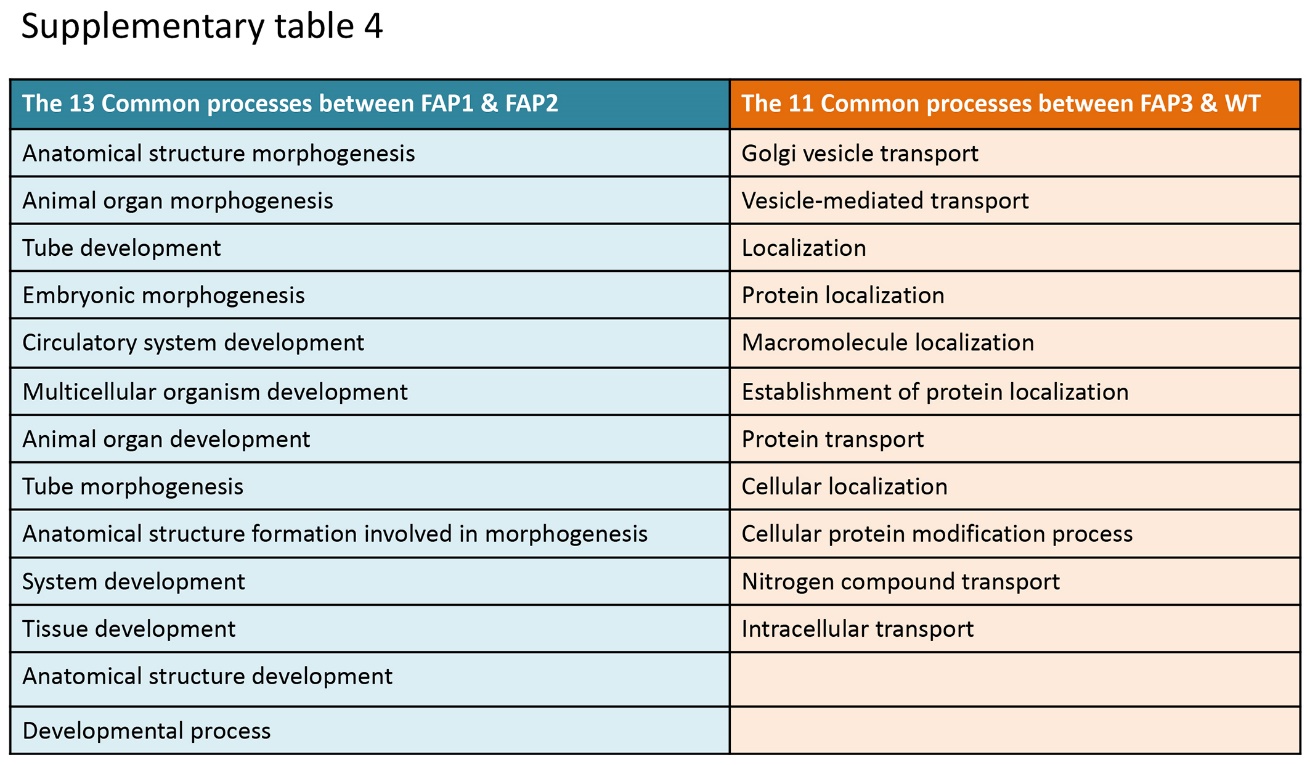
**
